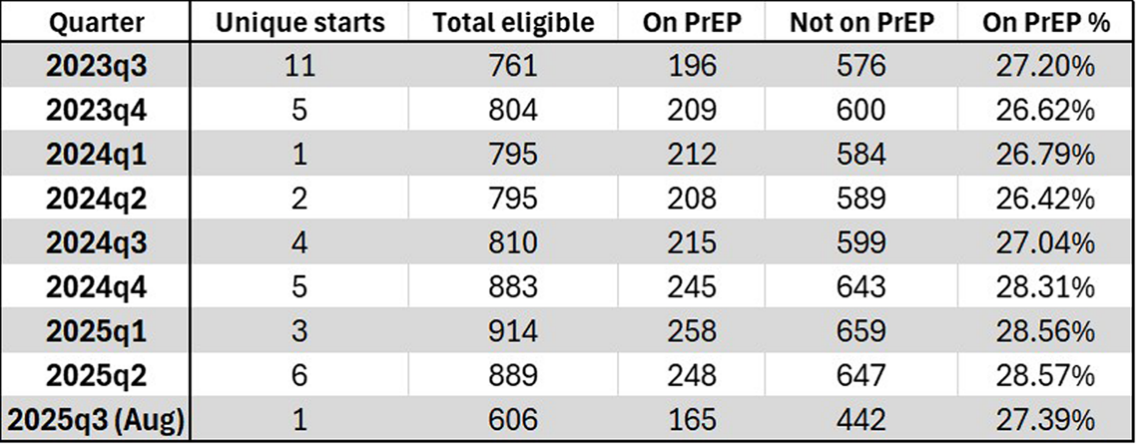# 202 Omission at Admission: A Retrospective Review of an Admission Surveillance Protocol for Candida auris

**DOI:** 10.1017/ash.2026.10590

**Published:** 2026-06-23

**Authors:** Jon Johannesson, Mary Townsend, Christopher Shoff, Maria Joyce, Reinaldo Perez

**Affiliations:** 1 Duke University Hospital; 2 Durham VA Medical Center; 3 Durham Veterans Affairs Healthcare System

## Abstract

**Background:** National guidelines recommend that anyone diagnosed with a sexually transmitted infection (STI) be tested for other STIs. However, in the majority of cases, appropriate STI co-testing is not performed. Additionally, STI diagnosis is often a missed opportunity for referral for HIV pre-exposure prophylaxis (PrEP). Order sets can be an effective tool within electronic medical records (EMR) to standardize diagnostic and therapeutic pathways. We sought to improve STI co-testing, PrEP referrals, and antimicrobial management through the implementation of a diagnostic and treatment order set within the Durham VA Health Care System (DVAHCS). **Methods:** An STI order set prompting co-testing was implemented in September of 2024 across DVAHCS. STI co-testing rates were analyzed one year pre- and post-implementation. Interrupted time series (ITS) analysis was performed using Gaussian regression to evaluate the effect of order set implementation for both co-testing of gonorrhea, chlamydia and syphilis (GCS) and additional cotesting for HIV (total STI). New starts and overall administration of HIV PrEP and order set utilization for therapeutics were analyzed using incidence rate ratios (IRRs) comparing incidence rates pre- and post-implementation. **Result:** The GCS cotesting IRR was 0.991 (p p p p p p p p p = 0.582). (Figure 3) The treatment order set was used on average 24.6% of the time after implementation. (Figure 4) **Conclusion:** The implementation of a STI co-testing order set did not change the rate of co-testing for chlamydia, gonorrhea, syphilis and HIV. An accompanying treatment order set was used to a significant degree, although in a minority of cases. There was no significant change in the initiation of PrEP after implementation of the order set as compared to prior. Our study was limited by small numbers of incident STI diagnoses. Further research is warranted to understand the optimal ways towards increasing recommended testing patterns and how tools within EMRs can assist with this goal.

**Figure:**
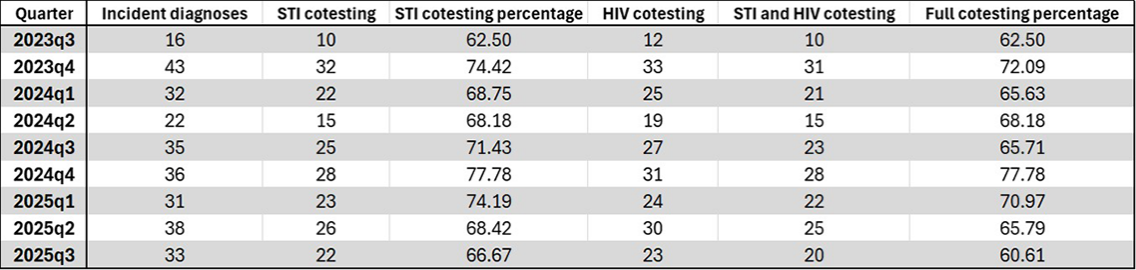

**Figure:**
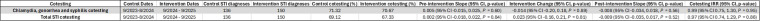

**Figure:**
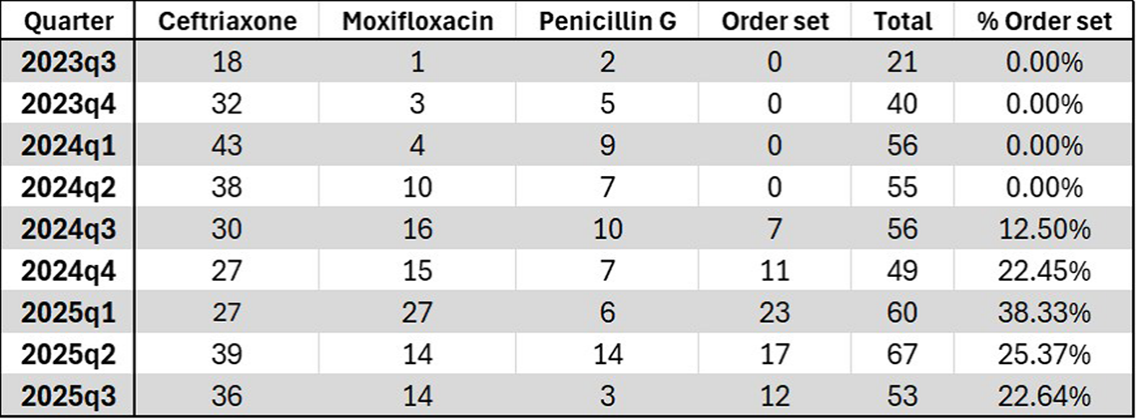

**Figure:**